# A bivalent vaccine confers immunogenicity and protection against *Shigella flexneri* and enterotoxigenic *Escherichia coli* infections in mice

**DOI:** 10.1038/s41541-020-0180-y

**Published:** 2020-03-27

**Authors:** Pedro Henrique Q. S. Medeiros, David T. Bolick, Solanka E. Ledwaba, Glynis L. Kolling, Deiziane V. S. Costa, Reinaldo B. Oriá, Aldo Ângelo M. Lima, Eileen M. Barry, Richard L. Guerrant

**Affiliations:** 1 Center for Global Health and Division of Infectious Diseases and International Health, University of Virginia, Charlottesville, VA USA; 2 Institute of Biomedicine, Federal University of Ceará, Fortaleza, CE Brazil; 3 Department of Microbiology, University of Venda, Thohoyandou, Limpopo province South Africa; 4 Center for Vaccine Development and Global Health, University of Maryland, Baltimore, MD USA

**Keywords:** Experimental models of disease, Preclinical research, Bacterial infection, Live attenuated vaccines

## Abstract

Vaccine studies for *Shigella flexneri* and enterotoxigenic *Escherichia coli* have been impaired by the lack of optimal animal models. We used two murine models to show that a *S. flexneri* 2a bivalent vaccine (CVD 1208S-122) expressing enterotoxigenic *Escherichia coli* colonization factor antigen-I (CFA/I) and the binding subunits A2 and B of heat labile-enterotoxin (LTb) is immunogenic and protects against weight loss and diarrhea. These findings document the immunogenicity and pre-clinical efficacy effects of CVD 1208S-122 vaccine and suggest that further work can help elucidate relevant immune responses and ultimately its clinical efficacy in humans.

## Introduction

*Shigella flexneri* and enterotoxigenic *Escherichia coli* (ETEC) are two major bacterial pathogens responsible for substantial burdens of diarrhea in children from developing countries^1,2^. Together they were responsible for more than 250,000 deaths in 2016 and about 20% of diarrhea deaths worldwide^3^.

Research conducted to enable development of effective and low-cost vaccines against *S. flexneri* and ETEC infections has been extensive over recent decades. However, there are still no licensed vaccines against these pathogens^4,5^. A multivalent approach targeting *Shigella* and ETEC concomitantly has been suggested to be ideal, due to lower costs, simpler delivery, and technical issues^4^.

The lack of small animal models that fully recapitulate clinical outcomes of infection has been one of the major barriers for development of *Shigella* and ETEC vaccines^5,6^. Recently, a combined *Shigella*–ETEC vaccine (CVD 1208S-122), consisting of an attenuated *S. flexneri* 2a strain expressing the ETEC colonization factor antigen-1 (CFA/1) and the heat labile-enterotoxin (LTb), has been shown to be safe and immunogenic in guinea pigs^7,8^. In order to provide stronger evidence of its pre-clinical effects, we evaluated whether CVD 1208S-122 could induce antibody responses and confer protection against diarrhea and weight loss using two murine models of orally administered *S. flexneri* and ETEC infections in C57BL/6 mice^9,10^. Following antibiotic treatment, these models enable the study of clinical outcomes that mimic human disease in children induced by oral infection and enable the testing of interventions. In this study, we used antibiotic pre-treated nourished mice as they closely mimic the disease most often seen in humans with optimal, self-limited diarrhea and weight decrements as reported^6,7^.

## Results

### Protection against disease and immunogenicity of CVD 1208S-122

**Fig. 1:**
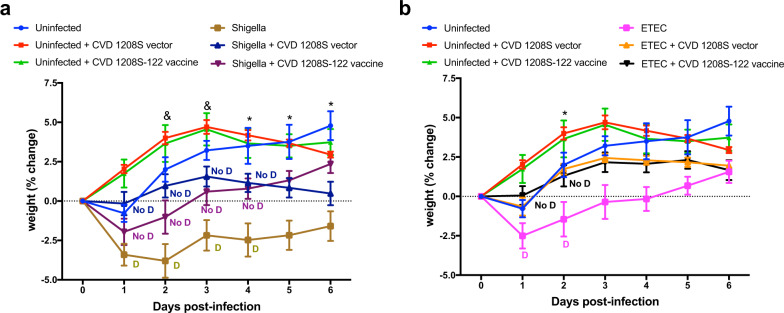
CVD 1208S-122 vaccine reduced weight loss and diarrhea in both *S. flexneri* 2457T and ETEC H10407 infected mice. Mice receiving CVD 1208S-122 vaccine or the vector were infected with either *Shigella flexneri* or enterotoxigenic *E. coli* (ETEC) and observed daily for bodyweight changes and diarrhea presence. **a** CVD 1208S-122 vaccine effects on bodyweight changes induced by *S. flexneri* during the first week of infection. *P < 0.05 by two-way ANOVA + Tukey’s test, Shigella vs Shigella+Vaccine at days 4, 5, and 6 post-infection; ^&^*P* < 0.05 by two-way ANOVA + Tukey’s test, Shigella vs Shigella + Vector at days 2 and 3 post-infection (two-way ANOVA *P* value < 0.001). **b** CVD 1208S-122 vaccine effects on bodyweight changes induced by ETEC during the first week of infection. *P < 0.05 by two-way ANOVA + Tukey’s test, ETEC vs ETEC + Vaccine at day 2 post-infection (two-way ANOVA *P* value < 0.001). Data are presented as mean with SEM. *N* = 16/group (*N* = 8 for Infected + Vector and *N* = 4 for Uninfected + Vector).

We first tested whether vaccination would be tolerated and prevent disease outcomes—weight loss and diarrhea—after infections with either *S. flexneri* 2457T or ETEC H10407 in mice fed a normal diet. Regarding protection against *S. flexneri* infection, the CVD 1208S-122-infected group showed a bodyweight change curve significantly higher than infected controls from days 4 to 6 post-infection (Fig. 1a). In addition, vaccination with CVD 1208S-122 prevented diarrhea on days 1, 2, 3, and 4 post-infection, as opposed to related *S. flexneri*-infected controls. Mice that received the live vector alone, CVD 1208S, were also protected against *Shigella* infection (days 2 and 3 post-infection).

Similarly, ETEC-infected mice that were pre-treated with CVD 1208S-122 did not show weight loss as compared with ETEC-infected controls, being significantly different on day 2 post-infection (Fig. 1b). Mice that received the vector control alone, CVD 1208S, showed a trend of protection against infection by ETEC, although not significant at day 2 post-infection. Diarrhea induced by ETEC infection was also prevented on days 1 and 2 post-infection by the CVD 1208S vector.

**Fig. 2:**
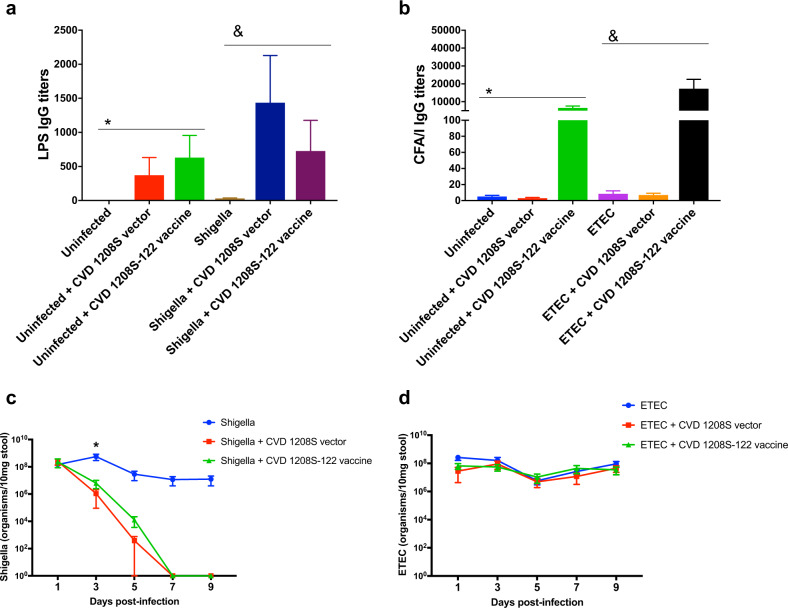
CVD 1208S-122 vaccine increased serum antibodies levels against both *S. flexneri* 2457T and ETEC H10407 and reduced *S. flexneri* stool shedding in infected mice. Mice receiving CVD 1208S-122 vaccine or the vector were infected with either *Shigella flexneri* or enterotoxigenic *E. coli* (ETEC). Fecal samples were collected daily for DNA extraction and further detection of the pathogens by real-time qPCR. Sera samples were collected at day 14 post-infection for antibody measurement by ELISA. **a** CVD 1208S-122 vaccine effects on anti-*Shigella* LPS IgG titers at day 14 post-infection. *P < 0.05 by Kruskal–Wallis + Dunn’s comparisons test, Uninfected vs Uninfected + Vector/Vaccine; ^&^*P* = 0.0013 and *P* = 0.0019 by Kruskal–Wallis + Dunn’s comparisons test, Shigella vs Shigella + Vaccine and Shigella vs Shigella + Vector, respectively. **b** CVD 1208S-122 vaccine effects on anti-ETEC CFA/I titers at day 14 post-infection. *P = 0.0001 by Kruskal–Wallis + Dunn’s comparisons test, Uninfected vs Uninfected + Vaccine; ^&^*P* < 0.0001 by Kruskal–Wallis + Dunn’s comparisons test, ETEC vs ETEC + Vaccine. **c** CVD 1208S-122 vaccine effects on stool shedding of *S. flexneri* in infected mice up to 9 days post-infection. *P < 0.05 by two-way ANOVA + Tukey’s test, Shigella vs Shigella + Vector/Vaccine (two-way ANOVA *P* value = 0.006). **d** CVD 1208S-122 vaccine effects on stool shedding of ETEC in infected mice up to 9 days post-infection. Data are presented as mean with SEM. *N* = 16/group (*N* = 8 for Infected + Vector and *N* = 4 for Uninfected + Vector).

We then tested whether the CVD 1208S-122 pre-treated mice that showed disease protection would also exhibit antibody production against *S. flexneri* and ETEC. We collected serum samples for antibody measurements from mice 2 weeks post-infection. Mice that received CVD 1208S-122 showed increased anti-*Shigella* LPS IgG titers in the serum compared to unvaccinated mice, regardless of infection with *S. flexneri* (proportion of response: 16/16 infected mice and 10/12 uninfected mice—as opposed to 0/14 of unvaccinated mice). Mice exposed to the vector also showed increased levels of anti-LPS antibodies compared to unvaccinated mice (8/8 infected mice and 4/4 uninfected mice). In addition, *S. flexneri* infection alone did not induce significant antibody production by day 14 post-infection (3/12 mice showed increased antibody production). Uninfected mice that received either the vector or the vaccine showed increased titers of anti-LPS in the serum. Further, CVD 1208S-122 significantly induced anti-LPS antibodies in *S. flexneri*-infected mice (Fig. 2a). There was no significant difference in antibody production between *S. flexneri*-infected mice that received CVD 1208S-122 or only the vector.

When analyzing anti-CFA/I antibody production, vaccinated mice showed higher anti-CFA/I IgG levels in the serum, regardless of infection with ETEC (proportion of response: 16/16 of infected mice and 12/12 of uninfected mice—as opposed to 0/14 of unvaccinated mice). In addition, ETEC infection alone did not induce significant antibody production by day 14 post-infection. Uninfected mice that received the vaccine showed higher levels of anti-CFA/I in the serum than non-vaccinated mice. CVD 1208S-122 also significantly induced anti-CFA/I antibody production in ETEC-infected mice (Fig. 2b).

After observing disease protection and immunogenicity by CVD 1208S-122 in both *S. flexneri* and ETEC infections, the mice were followed for stool shedding of these pathogens after inoculation. *S. flexneri* stool shedding was decreased in the vaccinated group at day 3 post-infection by approximately 3 logs (10^5^ vs 10^2^) (Fig. 2c). As expected, stool shedding was also reduced in vector CVD 1208S-vaccinated mice. With ETEC infection, stool shedding was not significantly different between vaccinated or non-vaccinated mice throughout at least 9 days post-infection (Fig. 2d).

## Discussion

In this study we evaluated the CVD 1208S-122 vaccine, which is a CFA/I and LTb expressing attenuated *S. flexneri* 2a strain. Serum IgG against *Shigella* LPS has been a good correlate of protection in *Shigella* vaccine studies^5^. Similarly, CFA/I is one of the most common ETEC colonization factors and has been used as a target for ETEC vaccine^11^. LTb is also a common antigen used in the vaccine studies^12^. Overall, the current findings expand our knowledge about the immunogenic potential of these antigens for further vaccine studies. In addition, we observed potential protection by the vector alone (attenuated *S. flexneri* strain) against ETEC-induced weight loss, which might be explained by heterologous immune response. A possible explanation for this phenomenon is the high similarity (80%) between EatA (from ETEC) and SepA (from *S. flexneri*), both serine protease autotransporters of the Enterobacteriaceae (SPATE), which can lead to cross-reactivity^13^.

Besides preventing weight loss and diarrhea from both infections, CVD 1208S-122 was able to decrease intestinal colonization of *S. flexneri*, but not of ETEC. Interestingly, CFA/I—one of the two ETEC components inserted in the CVD 1208S-122—is a major colonization factor in ETEC^14,15^. However, there is an increasing recognition that a greater variety of colonization factors play a role in colonization and pathogenesis of ETEC infections^16^. More specifically, it is possible that EtpA, an exoprotein adhesin of ETEC, can mediate adherence between flagella and intestinal cells^17^, leading to robust colonization even in vaccinated mice. Further, the protection against ETEC-induced disease outcomes in this model may have been facilitated by anti-LT antibody responses, not measured here. The capacity of CVD 1208S-122 to induce strong serum and mucosal antibody responses against the ETEC antigens expressed in the vaccine was recently reported in guinea pigs^7,8^ and will need to be evaluated in future studies utilizing these mouse models.

The potential of this vaccine for clinical use in preventing *S. flexneri* and ETEC infections in children clearly warrants further exploration. Moreover, the intranasal route here used is not ideal for this target population, hence oral CVD 1208S-122 immunization should be tested in future studies. In addition, the findings presented herein raise questions of whether this vaccine could be protective against other *Shigella* and ETEC serotypes. While we highlight that *S. flexneri* 2a contributes to a substantial burden of shigellosis worldwide and LT + CFA/I + ETEC strains are one of the most common types of ETEC infection in the clinics, further studies are underway to investigate potential cross-serotype protection.

In conclusion, our data show that CVD 1208S-122 is able to induce immune responses and protect against disease outcomes diarrhea and weight loss in C57BL/6 mice which are infected with either *S. flexneri* 2a or LT + ST + ETEC H10407. These observations support future clinical trials of this vaccine CVD 1208S-122 as well as show the value of these murine models in the assessment of other vaccine candidates.

## Methods

### Ethics statement

The current study was performed in accordance with the recommendations in the Guide for the Care and Use of Laboratory Animals of the National Institutes of Health. All relevant ethical regulations for animal testing and research were complied. The Committee on the Ethics of Animal Experiments of the University of Virginia approved the protocol (Protocol Number 3315), which is in accordance with the policies from the Institutional Animal Care and Use Committee of the University of Virginia (UVA). All efforts were made to minimize suffering of the animals.

### Animal husbandry

Weaned male 4-week-old C57BL/6 mice were purchased from Jackson Laboratories for all experiments. After arrival, mice were acclimated and fed a standard rodent diet (Harlan) ad libitum.

### CVD 1208S-122 vaccine and immunization protocol

A previous live attenuated derivative of *S. flexneri* 2a by deletion in the *guaBA* operon, *set* and *sen* genes^18^ (CVD 1208S) was used as a vector for constructing the *Shigella*–ETEC bivalent vaccine. The ETEC CFA/I-encoding operon and the LT A2 and B subunits were engineered into the chromosome of the CVD 1208S. This new bivalent vaccine (CVD 1208S-122) has been reported to be safe and immunogenic in guinea pigs^7,8,19^.

Six weeks prior to infection, mice received CVD 1208S-122 or the vector intranasally (10^6^ CFU/ mouse in 10 µL of saline) in three weekly doses. We used intranasal route due to vast evidence in the literature indicating its effective immunogenicity for shigellosis and other enteric infections^20,21^. In order to allow development of antibody production, we performed challenge infections 4 weeks after the last immunization.

### *S. flexneri* and ETEC infections

We performed oral inoculations of *S. flexneri* 2a strain 2457T or ETEC strain H01407 (refs. ^9,10^). Mice were previously exposed to a broad-spectrum antibiotic cocktail in the drinking water (metronidazole 215 mg/L, colistin 850 U/mL, gentamicin 35 mg/L, and vancomycin 45 mg/L) for 3 days. The antibiotic water was removed one day prior to infection. On the day of infection, bacteria were grown in a shaking culture in DMEM until media started to turn orange indicating maximum growth. Bacteria were centrifuged at 2739*g* at 4 °C and resuspended in fresh DMEM at a concentration allowing oral gavage of ~1 × 10^8^ bacteria for *S. flexneri* and ~1 × 10^9^ bacteria for ETEC in 100 µL. Uninfected controls were gavaged with 100 µL of DMEM as vehicle and gavage control.

### Quantitative analysis of *S. flexneri* and ETEC burdens

Fresh fecal pellets were obtained from individual mice, DNA extracted, and quantitative real-time PCR performed^9,10^. DNA was isolated from fecal pellets using the QIAamp DNA stool mini kit (Qiagen). Quantification of *S. flexneri* and ETEC were performed by real-time quantitative PCR. For *S. flexneri*, the protocol consisted of 3 min at 95 °C, followed by 40 cycles of 15 s at 95 °C, 60 s at 58 °C. The primer sequences used were: ipaH F 5′-ATGCGTGAGACTGAACAGCA-3′ and ipaH R 5′-GTGCAGTTGTGAGCCGTTTT-3′. For ETEC, the protocol consisted of 3 min at 95 °C, followed by 40 cycles of 10 s at 95 °C and 30 s at 58 °C. The primer sequences used were: LT F 5′-TTCCCACCGGATCACCAA-3′ and LT R 5′-CAACCTTGTGGTGCATGATGA-3′.

### Serum antibody measurements

Antibodies were measured in serum samples by ELISA. Antigens included hot water–phenol-extracted *S. flexneri* 2a LPS from strain 2457T and purified CFA/I^22^. To determine the end point titer, threefold dilutions of sera in 10% Blotto plus 0.05% Tween-20 were added to the coated plates and incubated overnight at 4 °C. Peroxidase-labeled secondary antibodies were developed with 3,3′,5,5′-tetramethylbenzidine (TMB) substrate for 15 min at room temperature and the OD_450_ was determined in an ELISA microplate reader (Multiscan Ascent; Thermo Labsystems, Helsinki, Finland). Serum samples were run in duplicate. Linear regression curves were plotted for each sample, and titers were calculated (through equaVOLtion parameters) as the inverse of the serum dilution that produces an OD_450_ of 0.2 above the blank. Mice that showed antibody titers at least 10 times higher than the baseline reflected by the mean of untreated mice were defined as responders.

### Statistical analysis

All experiments were conducted a minimum of two times and each graph shows measurements taken from distinct samples. Data presented in the graphs are either combined or representative of two experiments with similar results. Data collected was plotted an analyzed using GraphPad Prism software with two-sided tests. Two-way ANOVA tests followed by Tukey’s multiple comparisons tests were used for identifying significant differences between bodyweight changes and pathogen stool shedding across time among the groups, while Kruskal–Wallis tests followed by Dunn’s multiple comparisons tests were used for evaluating differences on antibody levels among the groups.

### Reporting summary

Further information on research design is available in the Nature Research Reporting Summary linked to this article.

## Supplementary information

Reporting summary

## Data Availability

All data generated during this study are available from the corresponding author upon request.
